# (2S*,3S*,3aS*,6S*,7aR*)-3-Hy­droxy-2-[(2R*,3S*)-3-isopropyl­oxiran-2-yl]-3,6-dimethyl-3,3a,5,6,7,7a-hexa­hydro-1-benzofuran-4(2*H*)-one

**DOI:** 10.1107/S1600536812038470

**Published:** 2012-09-19

**Authors:** Mingruo Ding, Qiaoyin Zhang, Lei Chen, Nianyu Huang, Junzhi Wang

**Affiliations:** a Hubei Key Laboratory of Natural Products Research and Development, College of Chemistry and Life Sciences, China Three Gorges University, Yichang, Hubei 443002, People’s Republic of China

## Abstract

In the title compound, C_15_H_24_O_4_, the six-membered ring shows a distorted chair conformation and the five-membered ring adopts an envelope conformation with the C atom bearing the methyl and OH groups as the flap. In the crystal, O—H⋯O hydrogen bonds link the mol­ecules into chains running along the *a*-axis direction.

## Related literature

The title compound was synthesized as a potential gastric cytoprotective agent. For background to gastric diseases, see: Palmer *et al.* (2010 ▶). For pharmacological uses of bis­abol­an­gelone, a sesquiterpene isolated from the roots of *Angelica polymorpha* Maxim, see: Fang & Liao (2006 ▶); Muckensturm *et al.* (1981 ▶). Huang *et al.* (2012 ▶); Wang *et al.* (2009 ▶). For the crystal structure of bis­abolangelone, see: Wang *et al.* (2007 ▶).
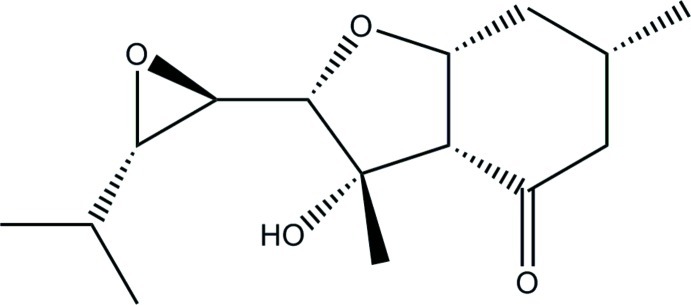


## Experimental

### 

#### Crystal data

C_15_H_24_O_4_
*M* *_r_* = 268.34
Orthorhombic, 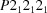

*a* = 6.616 (7) Å
*b* = 9.261 (9) Å
*c* = 25.12 (3) Å
*V* = 1539 (3) Å^3^
*Z* = 4
Mo *K*α radiation
μ = 0.08 mm^−1^
*T* = 296 K
0.35 × 0.28 × 0.26 mm

#### Data collection

Rigaku Mercury 375R CCD area-detector diffractometer
Absorption correction: multi-scan (*CrystalClear*; Rigaku, 2011 ▶) *T*_min_ = 0.972, *T*_max_ = 0.980
16467 measured reflections
2058 independent reflections
1568 reflections with *I* > 2σ(*I*)
*R*_int_ = 0.176

#### Refinement

*R*[*F* ^2^ > 2σ(*F* ^2^)] = 0.067
*wR*(*F* ^2^) = 0.185
*S* = 1.03
2058 reflections
177 parameters
H-atom parameters constrained
Δρ_max_ = 0.20 e Å^−3^
Δρ_min_ = −0.19 e Å^−3^

### 

Data collection: *CrystalClear* (Rigaku, 2011 ▶); cell refinement: *CrystalClear*; data reduction: *CrystalClear*; program(s) used to solve structure: *SHELXS97* (Sheldrick, 2008 ▶); program(s) used to refine structure: *SHELXL97* (Sheldrick, 2008 ▶); molecular graphics: *SHELXTL* (Sheldrick, 2008 ▶); software used to prepare material for publication: *SHELXTL*.

## Supplementary Material

Crystal structure: contains datablock(s) I, global. DOI: 10.1107/S1600536812038470/bt6831sup1.cif

Structure factors: contains datablock(s) I. DOI: 10.1107/S1600536812038470/bt6831Isup2.hkl

Supplementary material file. DOI: 10.1107/S1600536812038470/bt6831Isup3.cml

Supplementary material file. DOI: 10.1107/S1600536812038470/bt6831Isup4.cdx

Additional supplementary materials:  crystallographic information; 3D view; checkCIF report

## Figures and Tables

**Table 1:** Hydrogen-bond geometry (Å, °)

*D*—H⋯*A*	*D*—H	H⋯*A*	*D*⋯*A*	*D*—H⋯*A*
O3—H3*A*⋯O4^i^	0.82	2.02	2.827 (4)	166

Symmetry code: (i) 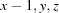.

**Fig. 1.**
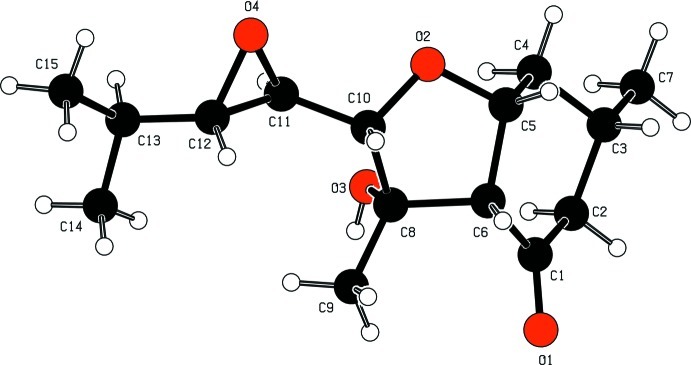
The molecular structure of the title compound with the atom numbering scheme. Displacement ellipsoids are drawn at the 50% probability level. H atom ise presented as small spheres of arbitrary radius.

**Fig. 2.**
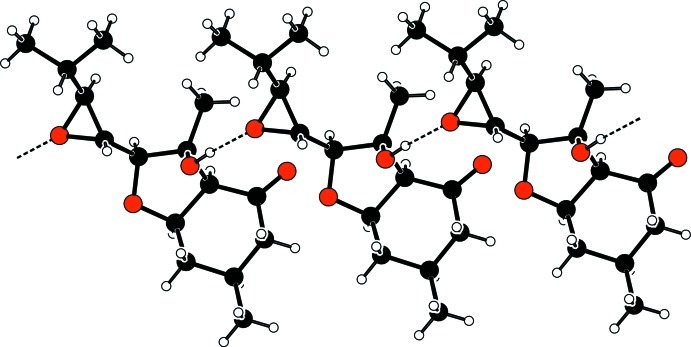
A packing diagram for the title compound showing O—H···O intra-molecular hydrogen bonds (dashed lines).

## supplementary crystallographic information

### Comment

Acid-related diseases are highly prevalent in the developed world, and the inhibition of the gastric proton pump enzyme (H^+^/K^+^-ATPase) represents a major approach in the development of drugs against these medical conditions (Palmer *et al.*, 2010). Bisabolangelone, a sesquiterpene isolated from the roots of *Angelica polymorpha* Maxim with the traditional Tujia medicine name of Zijinsha (Fang *et al.*, 2006), displayed attractive bioactivity such as anti-feeding and insecticidal effect (Muckensturm *et al.*, 1981). Recently, we found bisabolangelone and its derivatives also exhibited remarkably preventive and therapeutic action on gastric ulcer, and its anti-ulcer mechanism might be related to inhibition of the H^+^/K^+^-ATPase and reduction of the secretion of H^+^ (Wang *et al.*, 2009). With the aim of studying the relationship between its structure and H^+^/K^+^-ATPase inhibition activity, the catalytic hydrogenated reduction (Huang *et al.*, 2012) and epoxidation of bisabolangelone were undertaken, and the structure determination of the target compound was conducted by X-ray single-crystal analysis for the first time.

Compared with the crystal structure of bisabolangelone (Wang *et al.*, 2007), most bond lengths in the title compound are in the normal range of single or double bonds. The 6-membered ring C(1)—C(2)—C(3)—C(4)—C(5)—C(6) shows a distorted chair conformation [Φ = 319.3 (9)°, Θ = 146.4 (5)°, puckering amplitude (Q) = 0.485 (4)°]. The 5-membered ring O(2)—C(5)—C(6)—C(8)—C(10) adopts an envelope conformation with C(8) at the flap. Intermolecular O—H···O interactions link the molecules into infinite zigzag chains along the *a* axis, which contribute to the stability of the structure.

### Experimental

3-Hydroxy-3,6-dimethyl-2-(3-methylbut- 2-enylidene)-3,3a,7,7a-tetrahydrobenzofuran- 4(2*H*)-one (bisabolangelone, I, 1.00 g, 4.0 mmol) and Pd/C (0.10 g, 10% *w*/*w*) was dissolved in MeOH (30 ml) at 10\%C under dry nitrogen atmosphere, then hydrogen gas (99%) was bubbled into the vigorous stirred solution (50 ml/minute) for 2.0 h until the bisabolangelone was consumed. The hydrogen gas was diluted by large amounts of nitrogen and released into air through special pipeline, and the reaction mixture was filtered to recover the catalyst. Removing the solvents at reduced pressure to give white solids, which was purified by column chromatography on silica with ethyl acetate/petroleum ether (1:10, *v*/*v*) as eluent to give the pure intermediates 3-hydroxy-3,6-dimethyl-2-((*E*)-3-methylbut-1-enyl)hexahydrobenzofuran-4(2*H*)-one (II) as colorless needles (0.85 g). The *m*-CPBA (0.52 g, 1.5 mmol) and solid NaHCO_3_ (0.19 g, 2.5 mmol) were added to a solution of the intermediates II (0.25 g, 1.0 mmol) in dry CH_2_Cl_2_ (20 ml) at 0 °C. The solution was stirred for 10 h until complete consumption of the starting material. The reaction was quenched with saturated aqueous sodium thiosulfate solution and extracted with CH_2_Cl_2_ (3 × 15 ml). The combined organic extracts were washed with saturated aqueous NaHCO_3_ solution (25 ml) and dried over Na_2_SO_4_. The solventwas removed *in vacuo* and the residue purified by flash column chromatography on silica gel to give the pure (2*S*,3*S*,3*aS*,6*S*,7*aR*)-3-hydroxy-2-((2*R*,3*S*)-3-isopropyloxiran-2-yl)-3,6-dimethylhexahydrobenzofuran-4(2*H*)-one III (Eluant: ethyl acetate/petroleum ether = 1: 20, *v*/*v*). Single crystals suitable for X-ray diffraction were prepared by slow evaporation of a dilute solution of the title compound III in *n*-hexane:ethyl acetate, 10: 1 at room temperature.

### Refinement

Due to the absence of anomalous scatterers, the absolute configuration could not be determined and was arbitrarily set. Friedel pairs were merged. All H atoms were geometrically positioned and refined using a riding model with C—H = 0.93–0.97 Å and *U*_iso_(methyl H) = 1.5 *U*_eq_(C) and 1.2 *U*_eq_(C, O) for other H atoms. The methyl and hydroxyl group were allowed to rotate but not to tip.

### Crystal data

**Table:**

C_15_H_24_O_4_	*F*(000) = 584
*M**_r_* = 268.34	*D*_x_ = 1.158 Mg m^−^^3^
Orthorhombic, *P*2_1_2_1_2_1_	Mo *K*α radiation, λ = 0.71073 Å
Hall symbol: P 2ac 2ab	Cell parameters from 2058 reflections
*a* = 6.616 (7) Å	θ = 2.7–27.5°
*b* = 9.261 (9) Å	µ = 0.08 mm^−^^1^
*c* = 25.12 (3) Å	*T* = 296 K
*V* = 1539 (3) Å^3^	Prism, colorless
*Z* = 4	0.35 × 0.28 × 0.26 mm

### Data collection

**Table:**

Rigaku model name? CCD area-detector diffractometer	2058 independent reflections
Radiation source: fine-focus sealed tube	1568 reflections with *I* > 2σ(*I*)
Graphite monochromator	*R*_int_ = 0.176
phi and ω scans	θ_max_ = 27.5°, θ_min_ = 2.7°
Absorption correction: multi-scan (*CrystalClear*; Rigaku, 2011)	*h* = −8→8
*T*_min_ = 0.972, *T*_max_ = 0.980	*k* = −12→12
16467 measured reflections	*l* = −32→32

### Refinement

**Table:**

Refinement on *F*^2^	Primary atom site location: structure-invariant direct methods
Least-squares matrix: full	Secondary atom site location: difference Fourier map
*R*[*F*^2^ > 2σ(*F*^2^)] = 0.067	Hydrogen site location: inferred from neighbouring sites
*wR*(*F*^2^) = 0.185	H-atom parameters constrained
*S* = 1.03	*w* = 1/[σ^2^(*F*_o_^2^) + (0.0887*P*)^2^ + 0.2181*P*] where *P* = (*F*_o_^2^ + 2*F*_c_^2^)/3
2058 reflections	(Δ/σ)_max_ < 0.001
177 parameters	Δρ_max_ = 0.20 e Å^−^^3^
0 restraints	Δρ_min_ = −0.19 e Å^−^^3^

### Special details

**Table:**

Geometry. All e.s.d.'s (except the e.s.d. in the dihedral angle between two l.s. planes) are estimated using the full covariance matrix. The cell e.s.d.'s are taken into account individually in the estimation of e.s.d.'s in distances, angles and torsion angles; correlations between e.s.d.'s in cell parameters are only used when they are defined by crystal symmetry. An approximate (isotropic) treatment of cell e.s.d.'s is used for estimating e.s.d.'s involving l.s. planes.
Refinement. Refinement of *F*^2^ against ALL reflections. The weighted *R*-factor *wR* and goodness of fit *S* are based on *F*^2^, conventional *R*-factors *R* are based on *F*, with *F* set to zero for negative *F*^2^. The threshold expression of *F*^2^ > σ(*F*^2^) is used only for calculating *R*-factors(gt) *etc*. and is not relevant to the choice of reflections for refinement. *R*-factors based on *F*^2^ are statistically about twice as large as those based on *F*, and *R*- factors based on ALL data will be even larger.

### Fractional atomic coordinates and isotropic or equivalent isotropic displacement parameters (Å^2^)

**Table:**

	*x*	*y*	*z*	*U*_iso_*/*U*_eq_	
C1	0.3330 (6)	0.7529 (5)	0.08201 (17)	0.0809 (11)	
C2	0.2584 (5)	0.8871 (5)	0.05651 (16)	0.0771 (11)	
H2A	0.2052	0.9501	0.0840	0.093*	
H2B	0.1474	0.8623	0.0330	0.093*	
C3	0.4150 (6)	0.9702 (5)	0.02476 (14)	0.0788 (11)	
H3	0.4605	0.9093	−0.0048	0.095*	
C4	0.5947 (6)	1.0012 (5)	0.06053 (14)	0.0793 (11)	
H4A	0.6926	1.0586	0.0411	0.095*	
H4B	0.5500	1.0572	0.0910	0.095*	
C5	0.6938 (5)	0.8657 (5)	0.07967 (14)	0.0748 (11)	
H5	0.7693	0.8228	0.0501	0.090*	
C6	0.5481 (5)	0.7502 (4)	0.10272 (15)	0.0706 (9)	
H6	0.6049	0.6551	0.0944	0.085*	
C7	0.3260 (10)	1.1082 (7)	0.0018 (2)	0.1196 (19)	
H7A	0.2137	1.0847	−0.0208	0.179*	
H7B	0.4273	1.1577	−0.0185	0.179*	
H7C	0.2808	1.1692	0.0303	0.179*	
C8	0.5676 (5)	0.7732 (3)	0.16381 (14)	0.0626 (8)	
C9	0.5010 (8)	0.6450 (4)	0.1976 (2)	0.0937 (14)	
H9A	0.5276	0.6650	0.2344	0.141*	
H9B	0.5745	0.5605	0.1869	0.141*	
H9C	0.3589	0.6288	0.1927	0.141*	
C10	0.7945 (5)	0.8060 (4)	0.16665 (15)	0.0658 (9)	
H10	0.8702	0.7159	0.1621	0.079*	
C11	0.8628 (4)	0.8785 (4)	0.21676 (13)	0.0581 (8)	
H11	0.7903	0.9670	0.2264	0.070*	
C12	0.9471 (5)	0.7963 (4)	0.26096 (14)	0.0634 (8)	
H12	0.9520	0.6916	0.2554	0.076*	
C13	0.9347 (5)	0.8420 (4)	0.31751 (14)	0.0690 (9)	
H13	0.9259	0.9477	0.3180	0.083*	
C14	0.7449 (8)	0.7838 (6)	0.3432 (2)	0.1076 (17)	
H14A	0.6293	0.8128	0.3227	0.161*	
H14B	0.7335	0.8212	0.3787	0.161*	
H14C	0.7514	0.6803	0.3445	0.161*	
C15	1.1278 (9)	0.7993 (8)	0.3472 (2)	0.1161 (19)	
H15A	1.1347	0.6961	0.3501	0.174*	
H15B	1.1262	0.8411	0.3822	0.174*	
H15C	1.2434	0.8341	0.3280	0.174*	
O1	0.2253 (6)	0.6483 (5)	0.08748 (18)	0.1301 (15)	
O2	0.8347 (3)	0.8990 (3)	0.12242 (9)	0.0749 (7)	
O3	0.4646 (3)	0.9014 (2)	0.17901 (9)	0.0613 (6)	
H3A	0.3551	0.8803	0.1922	0.092*	
O4	1.0785 (3)	0.8795 (4)	0.22651 (11)	0.0802 (8)	

### Atomic displacement parameters (Å^2^)

**Table:**

	*U*^11^	*U*^22^	*U*^33^	*U*^12^	*U*^13^	*U*^23^
C1	0.074 (2)	0.087 (3)	0.081 (2)	−0.021 (2)	−0.003 (2)	−0.014 (2)
C2	0.0604 (18)	0.100 (3)	0.071 (2)	−0.010 (2)	0.0036 (16)	−0.015 (2)
C3	0.080 (2)	0.102 (3)	0.0548 (17)	−0.005 (2)	0.0107 (18)	−0.0113 (19)
C4	0.081 (2)	0.093 (3)	0.0630 (19)	−0.022 (2)	0.0109 (19)	0.004 (2)
C5	0.0617 (17)	0.106 (3)	0.0564 (17)	−0.009 (2)	0.0154 (16)	−0.022 (2)
C6	0.070 (2)	0.0609 (18)	0.081 (2)	−0.0009 (17)	0.0061 (18)	−0.0212 (17)
C7	0.133 (4)	0.135 (5)	0.090 (3)	−0.002 (4)	−0.015 (3)	0.025 (3)
C8	0.0639 (18)	0.0495 (15)	0.075 (2)	−0.0059 (15)	0.0072 (16)	−0.0049 (15)
C9	0.095 (3)	0.063 (2)	0.123 (4)	−0.020 (2)	−0.008 (3)	0.020 (2)
C10	0.0593 (17)	0.0631 (19)	0.075 (2)	0.0080 (16)	0.0098 (17)	−0.0040 (18)
C11	0.0485 (15)	0.0563 (17)	0.0695 (18)	0.0006 (14)	0.0097 (14)	0.0032 (15)
C12	0.0545 (16)	0.0559 (16)	0.080 (2)	0.0060 (15)	0.0053 (16)	0.0029 (16)
C13	0.074 (2)	0.0594 (17)	0.073 (2)	−0.0010 (17)	0.0024 (18)	0.0082 (16)
C14	0.129 (4)	0.096 (3)	0.098 (3)	−0.018 (3)	0.036 (3)	0.012 (3)
C15	0.121 (4)	0.128 (5)	0.099 (3)	0.023 (4)	−0.028 (3)	0.006 (3)
O1	0.124 (3)	0.114 (3)	0.153 (3)	−0.058 (2)	−0.045 (3)	0.012 (3)
O2	0.0550 (12)	0.104 (2)	0.0660 (13)	−0.0143 (14)	0.0110 (11)	−0.0024 (13)
O3	0.0545 (11)	0.0597 (12)	0.0696 (13)	−0.0028 (10)	0.0165 (10)	−0.0034 (11)
O4	0.0488 (11)	0.110 (2)	0.0821 (16)	−0.0093 (15)	0.0106 (11)	0.0071 (16)

### Geometric parameters (Å, º)

**Table:**

C1—O1	1.210 (6)	C9—H9A	0.9600
C1—C2	1.482 (7)	C9—H9B	0.9600
C1—C6	1.515 (6)	C9—H9C	0.9600
C2—C3	1.517 (6)	C10—O2	1.431 (4)
C2—H2A	0.9700	C10—C11	1.497 (5)
C2—H2B	0.9700	C10—H10	0.9800
C3—C4	1.518 (5)	C11—O4	1.448 (4)
C3—C7	1.520 (8)	C11—C12	1.457 (5)
C3—H3	0.9800	C11—H11	0.9800
C4—C5	1.495 (7)	C12—O4	1.448 (4)
C4—H4A	0.9700	C12—C13	1.484 (5)
C4—H4B	0.9700	C12—H12	0.9800
C5—O2	1.455 (4)	C13—C14	1.512 (6)
C5—C6	1.552 (6)	C13—C15	1.531 (6)
C5—H5	0.9800	C13—H13	0.9800
C6—C8	1.554 (5)	C14—H14A	0.9600
C6—H6	0.9800	C14—H14B	0.9600
C7—H7A	0.9600	C14—H14C	0.9600
C7—H7B	0.9600	C15—H15A	0.9600
C7—H7C	0.9600	C15—H15B	0.9600
C8—O3	1.420 (4)	C15—H15C	0.9600
C8—C9	1.525 (5)	O3—H3A	0.8200
C8—C10	1.533 (5)		
			
O1—C1—C2	121.6 (4)	C8—C9—H9A	109.5
O1—C1—C6	120.0 (5)	C8—C9—H9B	109.5
C2—C1—C6	118.4 (4)	H9A—C9—H9B	109.5
C1—C2—C3	115.2 (4)	C8—C9—H9C	109.5
C1—C2—H2A	108.5	H9A—C9—H9C	109.5
C3—C2—H2A	108.5	H9B—C9—H9C	109.5
C1—C2—H2B	108.5	O2—C10—C11	109.1 (3)
C3—C2—H2B	108.5	O2—C10—C8	105.4 (3)
H2A—C2—H2B	107.5	C11—C10—C8	115.1 (3)
C4—C3—C7	111.7 (4)	O2—C10—H10	109.1
C4—C3—C2	108.6 (3)	C11—C10—H10	109.1
C7—C3—C2	111.2 (4)	C8—C10—H10	109.1
C4—C3—H3	108.4	O4—C11—C12	59.8 (2)
C7—C3—H3	108.4	O4—C11—C10	116.3 (3)
C2—C3—H3	108.4	C12—C11—C10	121.5 (3)
C5—C4—C3	112.1 (4)	O4—C11—H11	115.8
C5—C4—H4A	109.2	C12—C11—H11	115.8
C3—C4—H4A	109.2	C10—C11—H11	115.8
C5—C4—H4B	109.2	O4—C12—C11	59.8 (2)
C3—C4—H4B	109.2	O4—C12—C13	116.9 (3)
H4A—C4—H4B	107.9	C11—C12—C13	124.0 (3)
O2—C5—C4	109.9 (3)	O4—C12—H12	114.9
O2—C5—C6	105.6 (3)	C11—C12—H12	114.9
C4—C5—C6	115.2 (3)	C13—C12—H12	114.9
O2—C5—H5	108.7	C12—C13—C14	110.6 (4)
C4—C5—H5	108.7	C12—C13—C15	110.3 (3)
C6—C5—H5	108.7	C14—C13—C15	113.1 (4)
C1—C6—C5	116.3 (4)	C12—C13—H13	107.5
C1—C6—C8	114.5 (3)	C14—C13—H13	107.5
C5—C6—C8	102.8 (3)	C15—C13—H13	107.5
C1—C6—H6	107.6	C13—C14—H14A	109.5
C5—C6—H6	107.6	C13—C14—H14B	109.5
C8—C6—H6	107.6	H14A—C14—H14B	109.5
C3—C7—H7A	109.5	C13—C14—H14C	109.5
C3—C7—H7B	109.5	H14A—C14—H14C	109.5
H7A—C7—H7B	109.5	H14B—C14—H14C	109.5
C3—C7—H7C	109.5	C13—C15—H15A	109.5
H7A—C7—H7C	109.5	C13—C15—H15B	109.5
H7B—C7—H7C	109.5	H15A—C15—H15B	109.5
O3—C8—C9	111.2 (3)	C13—C15—H15C	109.5
O3—C8—C10	107.0 (3)	H15A—C15—H15C	109.5
C9—C8—C10	114.3 (3)	H15B—C15—H15C	109.5
O3—C8—C6	109.9 (3)	C10—O2—C5	109.0 (3)
C9—C8—C6	114.7 (3)	C8—O3—H3A	109.5
C10—C8—C6	98.9 (3)	C11—O4—C12	60.4 (2)
			
O1—C1—C2—C3	−146.8 (5)	C9—C8—C10—O2	164.2 (3)
C6—C1—C2—C3	34.8 (5)	C6—C8—C10—O2	41.9 (3)
C1—C2—C3—C4	−54.8 (5)	O3—C8—C10—C11	48.0 (4)
C1—C2—C3—C7	−178.1 (4)	C9—C8—C10—C11	−75.6 (4)
C7—C3—C4—C5	−175.3 (4)	C6—C8—C10—C11	162.1 (3)
C2—C3—C4—C5	61.8 (4)	O2—C10—C11—O4	−76.3 (4)
C3—C4—C5—O2	−167.6 (3)	C8—C10—C11—O4	165.6 (3)
C3—C4—C5—C6	−48.5 (4)	O2—C10—C11—C12	−145.5 (3)
O1—C1—C6—C5	161.9 (4)	C8—C10—C11—C12	96.4 (4)
C2—C1—C6—C5	−19.6 (5)	C10—C11—C12—O4	104.2 (3)
O1—C1—C6—C8	−78.2 (6)	O4—C11—C12—C13	103.9 (4)
C2—C1—C6—C8	100.2 (4)	C10—C11—C12—C13	−152.0 (3)
O2—C5—C6—C1	148.0 (3)	O4—C12—C13—C14	160.0 (3)
C4—C5—C6—C1	26.6 (5)	C11—C12—C13—C14	89.8 (4)
O2—C5—C6—C8	22.0 (4)	O4—C12—C13—C15	−74.1 (4)
C4—C5—C6—C8	−99.4 (3)	C11—C12—C13—C15	−144.3 (4)
C1—C6—C8—O3	−53.2 (4)	C11—C10—O2—C5	−154.0 (3)
C5—C6—C8—O3	74.0 (3)	C8—C10—O2—C5	−29.9 (4)
C1—C6—C8—C9	73.1 (5)	C4—C5—O2—C10	129.2 (3)
C5—C6—C8—C9	−159.8 (3)	C6—C5—O2—C10	4.4 (4)
C1—C6—C8—C10	−164.9 (3)	C10—C11—O4—C12	−112.7 (4)
C5—C6—C8—C10	−37.8 (3)	C13—C12—O4—C11	−115.5 (3)
O3—C8—C10—O2	−72.2 (4)		

### Hydrogen-bond geometry (Å, º)

**Table:**

*D*—H···*A*	*D*—H	H···*A*	*D*···*A*	*D*—H···*A*
O3—H3*A*···O4^i^	0.82	2.02	2.827 (4)	166

Symmetry code: (i) *x*−1, *y*, *z*.
